# Machine learning and systems genomics approaches for multi-omics data

**DOI:** 10.1186/s40364-017-0082-y

**Published:** 2017-01-20

**Authors:** Eugene Lin, Hsien-Yuan Lane

**Affiliations:** 1 Graduate Institute of Biomedical Sciences, China Medical University, Taichung, Taiwan; 2 Vita Genomics, Inc, Taipei, Taiwan; 3 TickleFish Systems Corporation, Seattle, WA USA; 4 Department of Psychiatry, China Medical University Hospital, Taichung, Taiwan

**Keywords:** Genomics, Pharmacogenomics, Single nucleotide polymorphisms, Machine learning, Multi-omics, Systems genomics

## Abstract

In light of recent advances in biomedical computing, big data science, and precision medicine, there is a mammoth demand for establishing algorithms in machine learning and systems genomics (MLSG), together with multi-omics data, to weigh probable phenotype-genotype relationships. Software frameworks in MLSG are extensively employed to analyze hundreds of thousands of multi-omics data by high-throughput technologies. In this study, we reviewed the MLSG software frameworks and future directions with respect to multi-omics data analysis and integration. Our review was targeted at researching recent approaches and technical solutions for the MLSG software frameworks using multi-omics platforms.

## Background

Over the past few years, researchers and scientists have made remarkable progress in the interdisciplinary fields of precision medicine, data mining and predictive algorithms, bioinformatics, and computational medicine [1]. Machine learning and systems genomics (MLSG) approaches integrate multiple data types from multi-omics data by using data mining and predictive algorithms, pointing out that the MLSG approaches can support a more meaningful interpretation of phenotype-genotype relationships than an analysis using only a single data type. Therefore, there is an acute need for development of the MLSG software frameworks that can generate prediction of a given quantitative or categorical phenotype using next-generation multi-omic data [2].

Precision medicine, an emerging field of medicine, is becoming the cornerstone of medical practices with prospects of the customization of healthcare, which means medical decisions, practices, and treatments are tailored to individual patients [3]. The use of genomic biomarkers, such as multi-omics data, has played a major role in precision medicine in oncology and other chronic diseases such as asthma [4], mental disorders [5, 6], and diabetes [7–9]. More specifically, patients are divided into groups by genetic variability and other biomarkers so that medications may be tailored to individual patients with similar or related genetic characteristics [10, 11]. For example, accumulating evidence reveals that selected single nucleotide polymorphisms (SNPs) could be used as genetic markers to influence clinical treatment response and adverse drug reactions for antidepressants in patients with major depressive disorder [12–14]. With the advent of technology in multi-omics approaches such as genomics, proteomics, metabolomics, and epigenomics, we are able to employ materials or devices that can interact with biological systems at the molecular level and then target different molecules with high precision.

In big data science, machine learning methods are computer algorithms that can automatically learn to recognize complex patterns based on empirical data [15, 16]. The goal of an machine learning method is to enable an algorithm to learn from data of the past or present and use that knowledge to make predictions or decisions for unknown future events [17, 18]. In the general terms, the workflow for an machine learning method consists of three phases including build the model from example inputs, evaluate and tune the model, and then put the model into production in prediction-making. Some of the best-known algorithms in machine learning methods include naive Bayes [19], C4.5 decision tree [20], artificial neural networks (ANNs) [21–23], support vector machine (SVM) [24], k-Means [25], k-nearest neighbors (kNN) [26], and regression [27, 28]. There were some key emerging diagnostics studies for various diseases and treatments of significance for public health with consideration of machine learning methods, including applications in mental health [29–33], cancer [34–38], and pharmacogenetics [39–41].

In this review, we surveyed the MLSG software frameworks that could enable definite assessment of the phenotype-genotype interplay status by using multi-omics platforms. The MLSG software frameworks encompass the model-based integration (MBI), concatenation-based integration (CBI), and transformation-based integration (TBI) approaches (Table 1). Furthermore, we investigated some potential data reduction and feature selection approaches that can be leveraged together with the MLSG software frameworks. Finally, we summarized the future perspectives with respect to the MLSG approaches.

**Table 1:** Summary, strength, and limitation of each method of machine learning and systems genomics (MLSG) software frameworks

Software framework	Summary	Strength	Limitation
Model-based integration (MBI)	Multiple predictive models are generated by using various multi-omics data types; then a final predictive model is generated by using the multiple models.	Predictive models can be consolidated from various multi-omics data types, and each data type can be gathered from a various set of patients with same phenotype.	It may be challenging to avoid overfitting.
Concatenation-based integration (CBI)	Multiple data matrices of different multi-omics data types are incorporated into a large input matrix; then a predictive model is generated by using the large input matrix.	It is fairly easy to leverage various machine learning methods for analyzing continuous or categorical data once a large input matrix is formed.	It may be challenging to combine a large input matrix.
Transformation-based integration (TBI)	Datasets for various multi-omics data types are first converted into intermediate forms, which are united into a large input matrix; then a predictive model is generated by using the large input matrix.	Unique variables such as patient identifiers can be used to link multi-omics data types and integrate a variety of continuous or categorical data values.	It may be challenging to transform into intermediate forms.

## Model-based integration approach

**Fig. 1:**
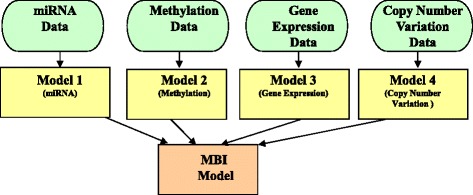
A flowchart for the model-based integration (MBI) software framework

First, we explored the MBI approach, which generates multiple models using different data types as training sets, and then generates a final model from the multiple models created during the training phase (Fig. 1). One advantage of the MBI approach is that this approach can merge predictive models from different data types and each data type can be assembled from a different set of patients with same phenotype [42].

In order to identify interactions between different levels of genomic data associated with certain disease or phenotype (for example, survival in ovarian cancer), the MBI approach can integrate multi-omics data, including, but not limited to, miRNA, methylation, gene expression, and copy number variation data. The MBI approach can then conduct the final multi-dimensional model from a particular machine learning algorithm (for example, Bayesian networks) with variables from the best models of each individual genomic dataset. Next, the MBI approach can compare the predictive power of the integration model with the one of the individual model from single level of genomic data to see whether the integration model can show the improvement. Finally, the MBI approach can obtain the best multi-dimensional model of all variables from multi-omics dimension as well as a balanced accuracy for the final model.

In the literature, the MBI approach encompasses the following computational frameworks for constructing a model: a majority voting approach [43], an ensemble classifier approach [44], and probabilistic causal networks [45]. In addition, we can employ the Analysis Tool for Heritable and Environmental Network Associations methodology, which is a suite of analysis tools for integrating multi-omics data [46].

## Probabilistic causal network framework

In order to integrate highly dissimilar types of data, we can leverage Bayesian networks that are one type of probabilistic causal networks [47]. Bayesian networks are directed acyclic graphs where the edges of the graph are represented by conditional probabilities, which define the distribution of states of each node given the state of its parents [47]. In Bayesian networks, each node characterizes a quantitative trait that can be a genomic factor (such as variation in DNA, gene expression, methylation, metabolite, and protein). These conditional probabilities represent not only relationships between genomic factors, but also the stochastic nature of these relationships. By assuming the observed data as a function of our prior belief, the Bayes formula is used to determine the likelihood of a Bayesian network model. Because the number of potential network structures grows super-exponentially with the number of nodes, it is infeasible to find the best model by an exhaustive search of all possible structures. Therefore, we can utilize Monte Carlo Markov Chain simulation [48] to pinpoint probably a huge amount of different plausible Bayesian networks, which are then integrated to accomplish a consensus network model. In the beginning, there is a null network. Then, slight arbitrary changes are made to the network by flipping, adding, or deleting individual edges. Ultimately, accepting those changes will lead to an overall improvement by fitting the network to the data. In order to avoid over-fitting owing to the addition of new parameters, the Bayesian information criterion score [49] can be employed to assess whether a change improves the network model.

## Ensemble classifier framework

In order to reduce the variance caused by the distinctiveness of a single genomic factor, Shen and Chou employed ensemble classifier models to integrate multiple classifiers, where each of those classifiers was based on individual genomic factor [50]. Thus, ensemble classifier models were able to obtain a more concrete concept in classification than a single classifier. The final output of the ensemble classifier model was the weighted fusion of the outputs generated by the individual basic classifiers. The weighted factor was assigned with the value of the success rate obtained by the individual basic classifier. Here, Shen and Chou adopted the optimized evidence-theoretic K-nearest-neighbors algorithm for the basic classifier [50].

## Concatenation-based integration approach

**Fig. 2:**
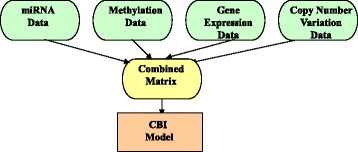
A flowchart for the concatenation-based integration (CBI) software framework

Second, we investigated the CBI approach, which combines multiple data matrices for each dataset into one large input matrix before constructing a model (Fig. 2). One advantage of the CBI approach is that, after we determine how to combine all of the variables into one matrix, it is relatively simple to employ a variety of machine learning methods for analyzing continuous or categorical data [42].

In the literature, the CBI approach encompasses the following computational frameworks for constructing a model: Bayesian networks [51], multivariate Cox LASSO models [52], grammatical evolution neural networks [53], iCluster [54], Bayesian correlated clustering [55], and Bayesian consensus clustering [56]. In addition, We can consider some of the best-known machine learning algorithms including naive Bayes [19], C4.5 decision tree [20], ANNs [21–23], SVM [24], k-Means [25], kNN [26], and regression [27, 28]. Depending on the number of variables in the data matrix, we can also employ data reduction and feature selection methods as described below.

In order to assess response to cancer therapeutics such as gemcitabine, Fridley et al. employed a Bayesian integrative model, which combines the ideas of Bayesian pathway analysis with Bayesian variable selection using stochastic search variable selection [51]. They employed two various high-throughput multi-omics datasets, such as mRNA expression and SNPs data, which were integrated into one large input matrix [51]. Fridley et al. reported that the Bayesian integrative model had greater sensitivity to detect genomic effects in the drug gemcitabine, as compared to the traditional single data type analysis [51].

Furthermore, instead of a single data type, Shen et al. implemented the iCluster framework to carry out cancer subtype discovery in glioblastoma using three multi-omics data types such as copy number data, mRNA expression data, and methylation data [54]. The iCluster framework is a CBI method that can simultaneously accomplish both data integration and dimension reduction to combine multi-omics data into one large input matrix [54]. Shen et al. revealed three distinct integrated tumor subtypes by using iCluster and multi-omics data [54].

### Transformation-based integration approach

**Fig. 3:**
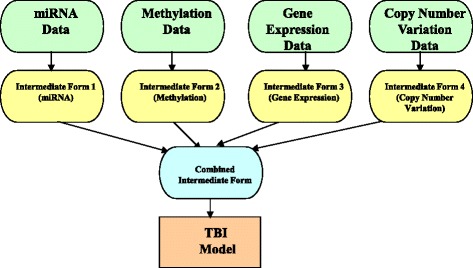
A flowchart for the transformation-based integration (TBI) software framework

Third, we assessed the TBI approach, which transforms each dataset into an intermediate form, such as a graph or a kernel matrix, and then merges multiple graphs or kernels into one before constructing a model (Fig. 3). One advantage of the TBI approach is that this approach can be employed to integrate a variety of continuous or categorical data values if the data contain unique variables such as patient identifiers for linking multi-omics data types [42].

In the literature, the TBI approach encompasses the following statistical frameworks for constructing a model: a kernel-based integration method [57] and a graph-based semi-supervised learning method [58]. The TBI approach investigates whether there is a relevant intermediate representation, such as a kernel or graph, for each multi-omics data type.

In order to find metabolic consequences underlying body weight change, Wahl et al. implemented a weighted correlation network approach [59], which was inferred using the Gaussian graphical model [60]. Instead of a single data type, they leveraged two different high-throughput multi-omics datasets, such as serum metabolomics and whole blood gene expression [59]. Wahl et al. first clustered multi-omics data into intermediate forms, namely modules of closely connected molecules, and then constructed a partial correlation network from the modules. Their analysis revealed that four metabolite and two gene expression modules were significantly associated with body weight change, indicating an association of long-term weight change with serum metabolite concentrations [59].

## Data reduction and feature selection approach

Accounting for models is not a trivial task because even a relatively small set of factors results in the large number of possible models [61]. For example, if we study 10 factors, then these 10 factors yield 2^10^ possible models. The purpose of data reduction and feature selection approaches is to find a subset of factors that maximizes the performance of the prediction model, depending on how these methods incorporate the feature selection search with the classification algorithms. There are two data reduction and feature selection approaches including extrinsic approaches (which use information external to the data set itself) and intrinsic approaches (which use the data set and some analytical technique for filtering). The extrinsic approaches, such as Biofilter [62], employ prior knowledge that is accessible in the public domain. Additionally, the intrinsic approaches encompass factor analysis [63], ReliefF [64], chi-square statistics, principal component analysis [65], and genetic algorithms [66].

Furthermore, a hybrid approach, which combines the information-gain method and the chi-squared method, is designed to reduce bias introduced by each of the methods [67]. Each feature is measured and ranked according to its merit in both methods. The measurement of the merit for the two methods is defined as follows. The information-gain method measures the decrease in the entropy of a given feature provided by another feature, and the chi-squared method is based on Pearson chi-squared statistic to measure divergence from the expected distribution. Next, all features are sorted by their average rank across these two methods. After the features are ranked, the classifiers are utilized to add one feature at a time based on its individual ranking and then select the desired number of the top ranked features that provides the best predictive performance, respectively.

Moreover, in a wrapper-based feature selection approach, the feature selection algorithm acts as a wrapper around the classification algorithm. The wrapper-based feature selection approach conducts best-first search for a good subset using the classification algorithm itself as part of the function for evaluating feature subsets [68, 69]. Best first search starts with empty set of features and searches forward to select possible subsets of features by greedy hill-climbing augmented with a backtracking technique [18].

## Future perspective

The MLSG modeling is essential to root out the false positive candidate genes discovered at the current association analyses by using meta-analysis, epistasis analysis, and pathway models [13]. Using multi-omics data not only could take care of missing information from any single data source, but also could help bridge the gap between phenotypes and more comprehensive biological regulation models [70]. In future research, models in MLSG will be established to predict the probability of drug efficacy to guide clinicians in choosing medications. In order to establish models for predicting drug efficacy, techniques in MLSG may provide a plausible way to predict drug efficacy in therapy. Finally, data analysis and integration in MLSG may play a key role in weighing gene–gene and gene–environment interactions.

## Conclusions

In this study, we reviewed several recent findings and relevant studies in terms of the MLSG software frameworks. The work also underscores the importance of techniques in MLSG to track down a greater diversity of populations in the clinical settings of diseases and their treatments. In fact, facilitating the MLSG tools based on multi-omics data plays a pivotal role, economically and clinically, in predicting the possible outcomes of diseases and treatments. Future research using the MLSG approaches is needed in order to weigh the interplay among clinical factors and multi-omics data.

## Abbreviations

ANNs: Artificial neural networks
CBI: Concatenation-based integration
kNN: k-nearest neighbors
MBI: Model-based integration
MLSG: Machine learning and systems genomics
SNPs: Single nucleotide polymorphisms
SVM: Support vector machine
TBI: Transformation-based integration
